# Single dose versus 24 h antibiotic prophylaxis in reduction mammaplasty: study protocol for a randomized controlled trial

**DOI:** 10.1186/s13063-020-04539-0

**Published:** 2020-07-02

**Authors:** Daniela Francescato Veiga, Edgard da Silva Garcia, José Wilson Moreira-Filho, Evelyne Borges de Mattos Andrade, Yara Juliano, Joel Veiga-Filho, Lydia Masako Ferreira

**Affiliations:** 1 Translational Surgery Graduate Program, Universidade Federal de São Paulo, São Paulo, Brazil; 2 Division of Plastic Surgery, Department of Surgery, Universidade do Vale do Sapucaí, Pouso Alegre, Brazil; 3 Department of Bioestatistics, Universidade Federal de São Paulo, and Universidade de Santo Amaro, São Paulo, Brazil

**Keywords:** Plastic surgery, Mammaplasty, Anti-bacterial agents, Prophylaxis, Wound infection

## Abstract

**Background:**

Reduction mammaplasty is among the most commonly performed procedures in plastic surgery. Antibiotics are widely prescribed, on an empirical basis, to prevent surgical site infections. However, there is a lack of evidence to support its use. This trial aims to compare the influence of the use of prophylatic antibiotics as a single dose or for 24 h on surgical site infection rates following reduction mammaplasty.

**Methods:**

Randomized trial of non-inferiority, with two parallel groups. A total of 146 breast hypertrophy patients, with reduction mammaplasty already scheduled, will be enrolled. Patients will be randomly allocated to the placebo group that will receive antibiotics only at the anesthesia induction (*n* = 73) or to the antibiotics group that will receive antibiotics at the anesthesia induction and for 24 h (*n* = 73). None of the patients will receive antibiotics after hospital discharge. Patients will be followed-up weekly, for 30 days, regarding surgical site infection. The Centers for Disease Control and Prevention criteria will be applied. A statistical analysis of the data will be performed.

**Discussion:**

Previous studies have demonstrated a decrease in infection rates after reduction mammaplasty when antibiotic prophylaxis was used, compared to the use of no antibiotics. However, the duration of antibiotic prophylaxis remains a point to be clarified. This study will test the hypothesis that maintaining the use of antibiotics for 24 h does not reduce infection rates compared to the use of a single preoperative dose.

**Trial registration:**

Clinicaltrials.gov NCT04079686. Registered on September 6, 2019.

## Administrative information

Note: the numbers in curly brackets in this protocol refer to SPIRIT checklist item numbers. The order of the items has been modified to group similar items (see http://www.equator-network.org/reporting-guidelines/spirit-2013-statement-defining-standard-protocol-items-for-clinical-trials/).

**Table:** 

Title {1}	Single dose versus 24 h antibiotic prophylaxis in reduction mammaplasty: study protocol for a randomized controlled trial
Trial registration {2a and 2b}.	Clinicaltrials.gov identifier: NCT04079686
Protocol version {3}	Version 2. June 25, 2019.
Funding {4}	This trial received grants from the Conselho Nacional de Desenvolvimento Científico e Tecnológico - CNPq (grant 302239/2018-9).
Author details {5a}	Daniela Francescato Veiga, MD, PhD^1,2^ - danielafveiga@gmail.com Edgard da Silva Garcia, MD, PhD^2^ - edgard.garcia@uol.com.br José Wilson Moreira Filho, MD, MSc^2^ - josemoreirafh@gmail.com Evelyne Borges de Mattos Andrade, MD^2^ - evelyne_andrade@hotmail.com Yara Juliano, PhD^3^ - yjuliano@prof.unisa.br Joel Veiga-Filho, MD, PhD^2^ - veigafilhoj@gmail.com Lydia Masako Ferreira, MD, PhD^1^ - lydiamferreira@gmail.com ^1^From Translational Surgery Graduate Program, Universidade Federal de São Paulo, São Paulo, Brazil ^2^From Division of Plastic Surgery, Department of Surgery, Universidade do Vale do Sapucaí, Pouso Alegre, Brazil ^3^From Department of Bioestatistics, Universidade Federal de São Paulo, and Universidade de Santo Amaro, São Paulo, Brazil Translational Surgery Graduate Program, Federal University of São Paulo, Rua Botucatu, 740, 2nd floor, Vila Clementino, São Paulo-SP, Brazil. CEP: 04023-062 Division of Plastic Surgery, Universidade do Vale do Sapucaí, Rua Comendador José Garcia, 777, Centro, Pouso Alegre-MG, Brazil. CEP: 37550-000
Name and contact information for the trial sponsor {5b}	Daniela Francescato Veiga Translational Surgery Graduate Program, Universidade Federal de São Paulo Rua Botucatu, 740 – 2nd Floor - Vila Clementino, São Paulo-SP E-mail: danielafveiga@gmail.com Phone number: 55-11-55764848 – Ext 3054
Role of sponsor {5c}	This trial is sponsored by the Translational Surgery Graduate Program - Universidade Federal de São Paulo, and it is coordinated by Prof. Daniela F. Veiga, as part of a research line. The trial received grants from the Conselho Nacional de Desenvolvimento Científico e Tecnológico - CNPq (grant 302239/2018-9). The coordinator and the research team are responsible for study design; collection, management, analysis, and interpretation of data; writing of the report; the decision to submit the report for publication, and they have ultimate authority over all of these activities. The funder has no authority over any of these activities.

## Introduction

### Background and rationale {6a}

Mammary hypertrophy is a frequent condition in women of different nationalities, and reduction mammaplasty is among the most commonly performed procedures in plastic surgery. According to the International Society of Aesthetic Plastic Surgery (ISAPS), in 2018, 534,294 reduction mammoplasties were performed worldwide, an increase of 8.5% compared with 2017 and 19.1% compared with 2014 [1]. Brazil is the country where this type of procedure is performed the most, with 98,900 reduction mammoplasties performed in 2018 [1]. Reduction mammaplasty has good long-term results, with a positive impact on different aspects of patient quality of life [2–7] and good cost-effectiveness [8].

The breast can be considered a surgical site that is clean or potentially contaminated [9, 10]. Reduction mammaplasty is generally classified as a clean surgery; however, infection rates are higher than those of other procedures in the same category [10–12]. Among the most frequent surgical complications of this procedure are healing disorders and surgical site infections (SSIs) [13, 14].

SSIs are defined as wound infections that occur following invasive surgical procedures [15–18]. They correspond to 14–16% of all nosocomial infections and are the most common among surgical patients [19].

The prevention of SSIs is extremely important due to the morbidity of SSIs, prolonged hospitalization times, and high costs [19–21]. Particularly in plastic surgery procedures, minimizing the risk of SSIs is imperative because even small infections are able to complicate the healing process and esthetic outcome [13, 14].

The use of antibiotics to prevent SSIs in plastic surgery is not clearly defined in the literature [12, 22, 23]. Nevertheless, antibiotic use has increased, especially in cosmetic procedures, in an effort to provide patients with higher safety standards [23, 24].

Authors have demonstrated a significant difference in SSI rates when antibiotic prophylaxis is used in reduction mammoplasties compared with the use of no antibiotic [13, 14, 25]. Shortt et al. performed a meta-analysis on antibiotic prophylaxis in reduction mammaplasty and found only three randomized clinical trials on the subject [11]. They observed a 75% reduction in SSIs compared with the use of placebo or no antibiotics, concluding that antibiotic prophylaxis is effective in preventing the occurrence of infection after reduction mammaplasty. However, they also emphasized the scarcity of data and the need for randomized clinical trials to evaluate the use of antibiotic prophylaxis in the postoperative period of reduction mammaplasty [11].

In 2013, the American Board of Plastic Surgery published the available evidence for reduction mammaplasty [26]. They presented data compiled since 2007 relating to reduction mammaplasty performed in 6451 patients in the USA by 606 plastic surgeons. Specifically, regarding the use of antibiotics, they found that 71% of patients received intraoperative antibiotics and 56% received antibiotics for more than 24 h. The systematic review presented in this same article concluded that the data available until that time supported the use of a single dose of antibiotics before the surgical incision, but there was a low level of evidence, and they stressed the need for more randomized clinical trials on the subject [26].

In January 2017, the American Board of Plastic Surgery published an update of the evidence in reduction mammaplasty [27]. Data obtained from 1343 plastic surgeons who performed reduction mammaplasty in 2010 patients in the USA showed that 98% of surgeons use antibiotics in the pre- and intraoperative periods and 58.2% of them maintain the use of antibiotics in the postoperative period [27].

In August 2017, the CDC published an update of the guidelines for SSI prevention [17]. A systematic review of the literature was conducted seeking the best evidence to support this new guideline. Specifically regarding the prophylactic use of antibiotics, they found that there was strong evidence that supports the use of antibiotics before making a surgical incision and indicated that the use of antibiotics in the postoperative period was not necessary in clean or potentially contaminated surgeries, even when drains were used [17]. However, there is a lack of evidence on the advantages or disadvantages of using more than one dose of antibiotics [18, 22].

Furthermore, the CDC guidelines are related to SSIs in general [17]. There is a lack of high-quality evidence specifically regarding reduction mammaplasty to support the practice. Considering that an infection can harm the esthetic result, which is important in a reduction mammaplasty, in addition to increasing costs and morbidity, many surgeons prefer to use antibiotics for a prolonged time [9, 25, 28, 29].

Recently, our group performed a clinical trial in which patients subjected to reduction mammaplasty received intravenous antibiotics during anesthetic induction and for 24 h. At hospital discharge, they were randomly allocated to a group that received an antibiotic prescription for 7 days or to a placebo group that received antibiotics only for 24 h after admission, and there was no significant difference between the groups, indicating that there is no need to maintain antibiotic administration over 24 h [30, 31]. However, as all the participants in both groups received antibiotics for 24 h, it was not clear if only one preoperative dose would suffice. The guidelines of the American Society of Plastic Surgeons (ASPS) for clinical practice in reduction mammaplasty also emphasized that although the administration of antibiotic prophylaxis decreases the infection rates after reduction mammaplasty, the lack of evidence does not allow a recommendation on the best timing of administration or the best duration of antibiotic use [22]. The present study will test the hypothesis that maintaining the use of antibiotics for 24 h does not reduce infection rates compared with the use of only one preoperative dose and may contribute evidence that may support clinical practice.

### Objectives {7}

This trial aims to compare the influence of the use of prophylatic antibiotics as a single dose or for 24 h on surgical site infection rates following reduction mammaplasty.

### Trial design {8}

A triple-blind (patients, surgical team, and outcome assessor) randomized non-inferiority clinical trial with two parallel groups with 1:1 allocation will be conducted.

## Methods: participants, interventions, and outcomes

### Study setting {9}

This study will be performed in a single center, promoted by the Translational Surgery Graduation Program—Universidade Federal de São Paulo and conducted at Samuel Libânio Clinics Hospital, a university hospital of the Universidade do Vale do Sapucaí located in Pouso Alegre, Minas Gerais, Brazil.

### Eligibility criteria {10}

A total of 146 patients with breast hypertrophy who are candidates for reduction mammaplasty who have scheduled surgeries, who meet the eligibility criteria for the study, and who sign a free and informed consent form will be consecutively selected at the outpatient clinics of Plastic Surgery at the Samuel Libânio Clinical Hospital. The following eligibility criteria will be considered:

Inclusion criteria—women aged between 18 and 60 years, those with a body mass index between 19 and 30 kg/m^2^, those with symptomatic breast hypertrophy, and those with a reduction mammaplasty already scheduled.

Exclusion criteria—previous restorative or esthetic procedure in the breasts, diagnosis of breast disease, smokers or those who had stopped smoking less than 1 month prior, parturition or lactation within the past year, comorbidities that constitute contraindications for the surgical procedure, patients who do not return to one of the weekly visits to evaluate the surgical wound, early surgical complications that require reintervention (e.g., hematoma), patients who receive antibiotic prescriptions for other reasons (infections of other body parts) during the follow-up period, or patients who withdraw their consent at any stage of the study.

### Who will take informed consent? {26a}

After the reduction mammaplasty is scheduled, one of the team surgeons will check whether the patient meets the eligibility criteria for the study. Those who meet the criteria will be informed about the study and invited to participate. Only those who agree to participate will be included after signing a free and informed consent form.

### Additional consent provisions for collection and use of participant data and biological specimens {26b}

This is not applicable as no participant data or biological specimens will be used in ancillary studies.

### Interventions

#### Explanation for the choice of comparators {6b}

Other authors demonstrated a significant difference in SSI rates when antibiotic prophylaxis is used in reduction mammoplasties compared with the use of no antibiotic [13, 14, 25]. In a previous study, patients undergoing reduction mammaplasty received intravenous antibiotics during anesthetic induction and for 24 h. At hospital discharge, they were randomly allocated to a group that received antibiotic prescription for 7 days or to a placebo group that received antibiotics only 24 h after admission, and there was no significant difference between the groups, indicating that there is no need to maintain antibiotic over 24 h [30, 31]. However, as all the participants in both groups received antibiotics for 24 h, it was not clear if only one preoperative dose would suffice. To try to clarify this issue, the current study will compare the effect of the use of antibiotics for 24 h with the use of only one preoperative dose on the infection rates after reduction mammaplasty.

#### Intervention description {11a}

All patients will undergo reduction mammaplasty at the Hospital Surgical Center of the Universidade do Vale do Sapucaí (University of Vale do Sapucaí). The surgeries will be performed under general anesthesia by the same surgical team led by a single surgeon. The conventional technique will be used with the resultant scar in an inverted “T” and a flap with superomedial pedicle for ascending the areola-papillary complex.

Patients will be hospitalized on the evening prior to the surgery and, before being taken to the operating room, will be instructed to take a shower with a 4% chlorhexidine solution [32]. Asepsis of the operating field will be achieved with a 0.5% alcohol solution of chlorhexidine [33].

All patients will receive 1 g of cefazolin via an intravenous route during the induction of anesthesia. At the end of the surgery, in the operating room, an envelope will be opened to determine the allocation of the patient to one of the two groups. Patients in the placebo group (*n* = 73) will be prescribed 100 ml of 0.9% sodium chloride every 6 h via the intravenous route, and for patients in the antibiotic group (*n* = 73), 1 g of cefazolin diluted in 100 ml of the same solution will be administered intravenously every 6 h during the hospitalization period. All patients will have to be discharged after 24 h.

#### Criteria for discontinuing or modifying allocated interventions {11b}

Patients who present adverse reactions to the use of antibiotics will have their administration immediately interrupted and will be monitored weekly for the occurrence of SSIs, according to the study protocol. Antibiotic therapy will be promptly instituted for patients who present SSIs during the follow-up period. Patients who have an early surgical complication (e.g., bruises) who require reintervention during the first 24 h will be excluded and may receive antibiotics at the discretion of the surgical team. Patients who receive antibiotics for other reasons (e.g., urinary or pulmonary infection) during the follow-up period will also be excluded from the study.

#### Strategies to improve adherence to interventions {11c}

A member of the surgical team will hold personal sessions with patients to emphasize the importance of adhering to medical recommendations. These sessions will be performed on the evening prior to the surgery, when the incisions are planned (markings with a pen on the skin), and at each weekly postoperative appointment. These sessions will include the following:

- Repeat the recommendations for postoperative care, and after asking if the patient has any questions, ask her to repeat the recommendations in her own words.
- Emphasize the importance of performing surgical wound care, wearing a bra, and avoiding physical activities.
- Ask about the possible occurrence of events that could constitute a criterion for exclusion from the study (e.g., use of antibiotics for other reasons).

#### Relevant concomitant care permitted or prohibited during the trial {11d}

Patients will be discharged after 24 h with prescription of analgesics (1 g of dipyrone orally every 6 h in case of pain). They will be instructed to wash the surgical wounds daily with soap and water in the shower and keep them dry and clean. They will also be instructed to return for weekly follow-up appointments, to wear a postoperative bra, to avoid physical activities, and not to use medications not prescribed by the surgical team during the 30 days of postoperative follow-up. Patients who need antibiotic therapy for other reasons (infections of other body parts) during the follow-up period will be excluded.

#### Provisions for post-trial care {30}

Patients will continue to be seen and followed up at the outpatient clinics of Plastic Surgery at the Samuel Libânio Clinical Hospital, free of charge, even after the end of the follow-up period.

### Outcomes {12}

The primary outcome is the occurrence of SSIs. The CDC considers SSIs that appear to be related to the surgical procedure if they occur up to 30 days after surgery in cases in which implants were not used or if they occur up to 1 year after surgery in cases in which implants were used [15].

To assess the occurrence of SSIs, patients will be evaluated once a week during the first 30 postoperative days because implants will not be used. The evaluation will be performed by a single surgeon who is a senior member of the surgical team with extensive experience in mammary surgeries and who will not know the allocation of the patients. This surgeon will use the criteria and classifications of the CDC for SSIs [15] and will dichotomize the outcome as the occurrence of an SSI (Yes/No). When there is an SSI, the same surgeon will classify the occurrence as a superficial incisional SSI, a deep incisional SSI, or an organ/space SSI, according to the CDC criteria [15].

### Participant timeline {13}

**Fig. 1:**
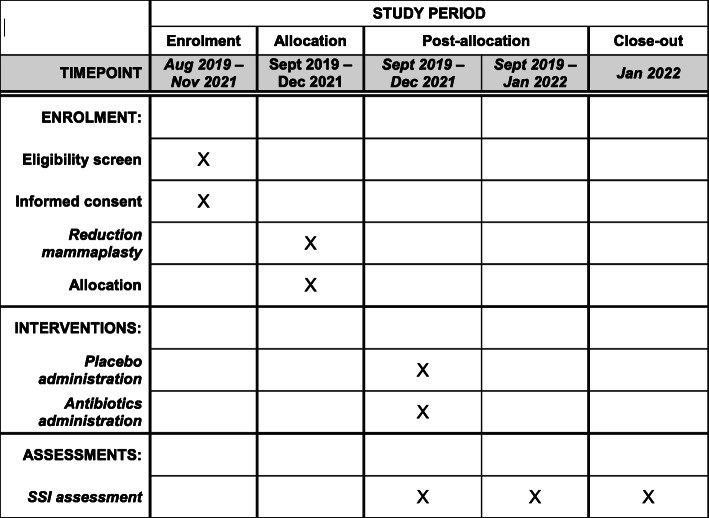
Time schedule of enrolment, interventions, and assessments for the participants of the trial

Figure 1 presents the time schedule of enrolment, interventions, and assessments for the participants of the trial.

### Sample size {14}

The sample size was calculated with a test power of 80%, single-tailed, with a significance level of 5%. To compare two proportions, the results of the meta-analysis published by Shortt et al., who found a 10% SSI rate when only preoperatively administered antibiotics [11], and the results of the clinical trial of Garcia et al., who observed a global SSI rate of 0.8% when antibiotics were administered preoperatively and maintained for 24 h [31], were considered. The calculation resulted in 73 patients per group.

### Recruitment {15}

Patients will be selected at the outpatient clinics of Plastic Surgery at the Samuel Libânio Clinical Hospital among those patients who already have a reduction mammaplasty scheduled by the Unified Public Health System (Sistema Único de Saúde—SUS). Because breast hypertrophy is a frequent condition and reduction mammaplasty will be performed by the SUS at no cost to the patients, the demand for this surgery is high; therefore, there is no expectation of difficulty in recruiting patients for this research.

## Assignment of interventions: allocation

### Sequence generation {16a}

Patients will be allocated to the placebo or antibiotic groups at a ratio of 1:1 based on a random sequence generated by the program Bioestat 5.3 (Mamirauá Institute, Brazil).

### Concealment mechanism {16b}

Allocation confidentiality will be maintained by the use of sequentially numbered opaque sealed envelopes prepared and stored by the surgeon who generated the sequence. The envelope will be opened after the end of the procedure always by a member of the surgical team (who did not generate the sequence, prepare the envelopes, or perform the evaluation of the outcome), and this member will make the postoperative prescription of cefazolin or saline solution, as previously described. The prescription will be made electronically by means of a password that can only be accessed by the nursing team in the sector where the patients will be hospitalized.

### Implementation {16c}

The generation of the allocation sequence will be performed by a single member of the surgical team who will not participate in the recruitment of patients or evaluation of the outcome. The numbered envelopes will be prepared and kept by this surgeon during the study. At the end of the surgical procedure, the envelope will be opened by another member of the surgical team who will make the prescription of a placebo or antibiotic according to the given allocation.

## Assignment of interventions: blinding

### Who will be blinded {17a}

The patients, the surgeons responsible for the recruitment, the surgical team, the surgeon performing the weekly outcome evaluation (SSI), and the biostatistician who will analyze the data will be blinded to the allocation of patients to the groups. A single surgeon who will prescribe the antibiotic or placebo will know the allocation. This surgeon will not participate in the recruitment or evaluation of the outcome.

The prescription will only be accessible by the nursing team in the sector where the patients will be admitted. The nursing team will receive training to prepare the solution for both groups in identical flasks with 100 ml of saline solution, which will be identified for administration only as “study solution,” in addition to the patient data, according to the hospital protocol.

### Procedure for unblinding if needed {17b}

Patients who present with adverse drug reactions within 24 h of hospitalization will have the confidentiality of the allocation broken. The prescription will be made available to the attending physician for clarification and immediate institution of appropriate therapy.

## Data collection and management

### Plans for assessment and collection of outcomes {18a}

The researchers will collect demographic and clinical data from each patient in a standardized form at the time of inclusion in the study. In this same form, data regarding the surgery such as surgical time, resected mammary tissue weight, and eventual complications will be recorded. Weekly data on the occurrence of SSIs will also be collected with a standardized form.

### Plans to promote participant retention and complete follow-up {18b}

Interventions will be performed during hospitalization, which will facilitate retention. At hospital discharge, the importance of following the care recommended by the medical team and attending weekly visits for follow-up will be emphasized (the patient will receive a card with the date, time, and location of each of the four postoperative visits planned). On the evening prior to each weekly appointment, the patient will be contacted by phone to be reminded.

### Data management {19}

All data collected during the study will be tabulated in a single spreadsheet to maintain allocation blinding and confidentiality. At the end of the study, after the last included patient undergoes surgery and the follow-up period is completed, the allocation group of each patient will be revealed, and separate worksheets will be prepared for the placebo and antibiotic groups. These spreadsheets will be sent for statistical analysis with groups designated as A and B to maintain the blinding of the biostatistics.

### Confidentiality {27}

All personal information of the participants will be kept in files protected by passwords with limited access. These files will be named by using the protocol number of each patient. In the spreadsheets that will be subjected to statistical analysis, the patients will also be identified only by their protocol numbers.

### Plans for collection, laboratory evaluation, and storage of biological specimens for genetic or molecular analysis in this trial/future use {33}

This is not applicable as no biological specimens were collected as part of this trial.

## Statistical methods

### Statistical methods for primary and secondary outcomes {20a}

The analysis will be performed with the program Bioestat, version 5.3 (Mamirauá Institute, Brazil), and the level of rejection of the null hypothesis will be set at 5%. For the numerical variables, descriptive statistics will be used, with calculations of the median, mean, and standard deviation. The Mann-Whitney test will be used to compare the groups regarding age, BMI, duration of the surgical procedure, and weight of resected breast tissue.

The chi-square test or Fisher’s exact test will be used to compare the groups with respect to the occurrence of SSIs.

### Interim analyses {21b}

As the patients in both groups will be monitored weekly and antibiotic therapy will be immediately instituted in case of infection diagnosis, no interim analysis is planned in this study.

### Methods for additional analyses (e.g., subgroup analyses) {20b}

Patients in each group will be stratified by age, BMI, duration of surgery, and weight of resected breast tissue (subdivided as below or above the median). The chi-square test or Fisher’s exact test will be used to compare the subgroups with respect to the occurrence of SSIs.

### Methods in analysis to handle protocol non-adherence and any statistical methods to handle missing data {20c}

Data analysis will be performed based on the original allocation of all patients, as defined by the randomization, regardless of the degree of adherence to the protocol (intention-to-treat principle). With regard to the missing data, the quantity, patterns, and variables associated with the omission will define the most appropriate technique to be used in the processing of these data. For primary analyses, methods that make use of all available data, such as multiple imputations, will be used.

### Plans to give access to the full protocol, participant-level data, and statistical code {31c}

Access to the full protocol and partial data sheet can be obtained from the principal investigator upon reasonable request.

## Oversight and monitoring

### Composition of the coordinating center and trial steering committee {5d}

The Coordinating Center is the Postgraduate Program in Translational Surgery of the Federal University of São Paulo. The Steering Committee is composed of two plastic surgeons, senior researchers at the Coordinating Center, one of whom is also linked to the University of Vale do Sapucaí, where data from the study will be collected. The Steering Committee will be responsible for the coordination and supervision of all stages of the study.

### Composition of the data monitoring committee, its role and reporting structure {21a}

Given that this is a non-inferiority study that involves interventions based on the literature and widely used in clinical practice, the establishment of a data monitoring committee was considered unnecessary.

### Adverse event reporting and harms {22}

In addition to the weekly medical consultations during the first 30 postoperative days, all patients will be instructed to contact a member of the surgical team (the patients will be told at least one member of the team at the hospital) if they have any problems related to surgery. They will also be instructed to contact this individual if they have any clinical complications, even if unrelated to surgery, that require medical evaluation.

### Frequency and plans for auditing trial conduct {23}

This is not applicable as external trial conduct audits are not planned for this trial.

### Plans for communicating important protocol amendments to relevant parties (e.g., trial participants, ethical committees) {25}

Any relevant modifications of the protocol, including changes in the study objectives, study design, eligibility criteria, interventions, or significant administrative aspects, among others, will require a formal alteration of the protocol. This type of modification will be agreed upon by the researchers and submitted to the approval of the Research Ethics Committee prior to its implementation.

### Dissemination plans {31a}

After completion, the full study will be published according to CONSORT 2010. The results should be presented at plastic surgery conferences for dissemination among physicians who work in the area. Patients will also be informed of the end of the study and will have access to the main results.

## Discussion

Reduction mammaplasty was the eighth most commonly performed plastic surgery in the world in 2018 [1]. Scarring problems are common in reduction mammaplasty and range from minor problems such as marginal skin necrosis, often seen at the junction of the inverted “T” incision, to important infections that require surgical reintervention [23]. Many surgeons prefer to administer antibiotics, believing that this will reduce the incidence of these problems [25].

Although it is generally considered a clean surgery, reduction mammaplasty has higher infection rates than other procedures in the same category [11, 14, 34]. SSIs, along with healing problems, are among the most common postoperative complications of reduction mammaplasty [11, 13, 31].

The risk factors and preventive measures for SSIs have not been well studied for ethical or logistical reasons. In a literature review, Junker et al. observed the time at which antibiotic prophylaxis was administered and the occurrence of intraoperative perforation of the gloves as factors that significantly influenced the occurrence of SSIs. Other evaluated factors, such as anemia, blood transfusion, and the surgeon’s experience, did not significantly influence the occurrence of SSIs [19].

The use of antibiotic prophylaxis in mammary surgeries is controversial in the literature. Although there are authors who prefer not to use antibiotics [34], studies and reviews have shown benefits in the administration of prophylactic antibiotics, especially before the surgical incision is made [13, 14, 19, 22, 25–27, 35].

Specifically, regarding reduction mammaplasty, Ahmadi et al., Veiga-Filho et al., and Vieira et al. demonstrated a reduction of SSI rates after reduction mammoplasties when antibiotic prophylaxis was used compared with the use of no antibiotic [13, 14, 25]. However, the duration of antibiotic prophylaxis remains to be clarified [11, 12, 22].

Many surgeons opt to maintain the use of antibiotics in the postoperative period. Data from the American Board of Plastic Surgery showed that more than half of the investigated North American plastic surgeons maintained the use of antibiotics for more than 24 h after reduction mammaplasty [26, 27].

A clinical trial recently completed by our group did not find a significant difference in the SSI rates after reduction mammoplasties when antibiotics were used for 24 h or for 7 days postoperatively [31]. However, as all the participants in both groups received antibiotics for 24 h, it was not clear if only one preoperative dose would suffice. The results of the present study may clarify this issue and provide evidence to support clinical practice.

### Trial status

Protocol version 2 (June 25, 2019). This trial is recruiting. The first patient was randomized in August 2019. By March 2020, 52 patients had undergone reduction mammaplasty. Three of these patients had SSI, diagnosed on the second postoperative week. All of them had undergone appropriate antibiotic therapy, with complete remission of the condition. Recruitment is expected to be completed by November 2021.

## Data Availability

The datasets used and/or analyzed during the current study are available from the corresponding author on reasonable request.

## Abbreviations

BMI: Body mass index
CDC: Centers for Disease Control and Prevention
SSI: Surgical site infection
